# Mosaic AAV mediates efficient transduction across the central and peripheral nervous systems

**DOI:** 10.1186/s13041-025-01270-2

**Published:** 2025-12-28

**Authors:** Min Jiang, Wenqing Yin, Xiaolu Mo, Zhounan Wang, Mengsheng Qiu, Zhong-Min Dai

**Affiliations:** Key Laboratory of Organ Development and Regeneration of Zhejiang Province, College of Life and Environmental Sciences, Hangzhou Normal University, Hangzhou, 311121 People’s Republic Of China

**Keywords:** AAV-PHP.eB, AAV-PHP.S, CNS, PNS

## Abstract

Adeno-associated virus (AAV) is a promising vector for neurological gene therapy, yet engineered serotypes are restricted to targeting either the central or peripheral nervous system (CNS or PNS). To overcome this limitation, we generated AAV with mosaic capsid, AAV-PHP.(S + eB), by co-packaging the AAV with two engineered capsid variants: AAV-PHP.eB and AAV-PHP.S, which exhibits strong CNS tropism and PNS tropism, respectively. Systemic administration of AAV-PHP.(S + eB) in adult mice mediated widespread transgene expression throughout the CNS, comparable to AAV-PHP.eB, while simultaneously achieving robust transduction of dorsal root ganglia neurons, similar to AAV-PHP.S. Notably, the mosaic vector demonstrated significantly reduced off-target transduction in the liver compared to both parental vectors, suggesting an improved safety. These results indicate that mosaic capsid assembly is a potent strategy for designing dual-tropic AAV vectors without increasing viral dose. This approach holds significant promise for treating complex neurological disorders that involve both nervous system compartments.

## Introduction

 Adeno-associated virus (AAV) is a promising tool for the treatment of neurological diseases due to its low immunogenicity and ability to provide stable, long-term transgene expression in non-dividing cells [1, 2]. One of the most significant challenges in the treatment of neurological diseases is how to effectively deliver gene therapy vectors to the central nervous system (CNS) [3]. The blood-brain barrier (BBB) restricts the passage of most gene gene delivery vectors from the bloodstream into the brain [4]. While most natural AAV serotypes have limited permeability across the BBB, AAV9 has been demonstrated to be more effectively transduce the CNS through systemic administration routes [5, 6]. However, gene delivery mediated by AAV9 requires a high viral load to achieve relatively limited transduction in the CNS [7–9]. AAV-PHP.eB, a novel engineered AAV serotype derived from AAV9 [5, 10], significantly enhanced CNS transduction efficiency and broader cellular coverage, making it a promising vector for treating neurological disorders.

Targeted AAV delivery has become increasingly prevalent not only in the CNS but also in the PNS, with significant advancements in gene delivery to the PNS in recent years. Among various AAV serotypes, AAV-PHP.S, a variant derived from AAV9, has demonstrated exceptional transduction efficiency within the PNS [5]. AAV-PHP.S facilitates efficient transduction of peripheral neurons primarily via intravenous injection, with its transduction profile encompassing key peripheral neural structures such as the dorsal root ganglia (DRG), cardiac ganglia, and the enteric nervous system (ENS) [10–12]. These properties render AAV-PHP.S a valuable tool for studying the underlying mechanisms of PNS disorders and for the development of gene therapy approaches.

CNS and PNS are structurally and functionally interconnected, influencing each other in various ways. Many CNS disorders are often associated with PNS-related issues [13], while diseases of the PNS can also impact the CNS [14], Most AAV vectors, due to their structural characteristics and receptor-binding properties, are limited to targeting either the CNS or PNS exclusively [15]. However, through genetic engineering and directed evolution techniques, several AAV variants, including AAV-MaCPNS1, AAV-MaCPNS2 [16] and AAV-rh10 [17], have been developed, demonstrating varying degrees of dual-targeting ability for both the CNS and PNS. Achieving efficient dual-system transduction, however, typically requires higher viral vector doses, which inevitably lead to increased liver toxicity [18].

Here, we report highly efficient transduction of both the CNS and PNS by the mosaic AAV-PHP.(S + eB), generated via co-packaging of Rep/Cap plasmids encoding AAV-PHP.eB and AAV-PHP.S. Systemic administration of AAV-PHP.(S + eB) in adult mice resulted in widespread transgene expression across diverse CNS regions while maintaining robust transduction of dorsal root ganglion (DRG) neurons, comparable to AAV-PHP.S. Importantly, AAV-PHP.(S + eB) exhibited lower hepatic transduction relative to either parental capsid, suggesting an improved safety profile for systemic delivery. Collectively, these findings establish AAV-PHP.(S + eB) as a dual-tropic vector with the potential to facilitate comprehensive gene therapy strategies targeting both central and peripheral components of the nervous system.

## Result

### Mosaic AAV-PHP.(S + eB) mediates efficient transduction across the CNS after intravenous injection

Two AAV variants with distinct tissue tropisms, AAV-PHP.eB and AAV-PHP.S, have been previously reported [10]. While AAV-PHP.eB mediates efficient transduction in the CNS, AAV-PHP.S exhibits strong tropism for the PNS. Based on the 60-mer structure of the AAV capsid, we hypothesized that mosaic AAV capsids composed of both AAV-PHP.eB and AAV-PHP.S would exhibit dual transduction capacity. To test this hypothesis, we co-transfected 293T cells with Rep/Cap plasmids encoding AAV-PHP.eB and AAV-PHP.S (1:1 ratio), together with a CMV-EGFP AAV reporter plasmid and helper plasmid. The resulting recombinant virus was designated AAV-PHP.(S + eB).

**Fig. 1:**
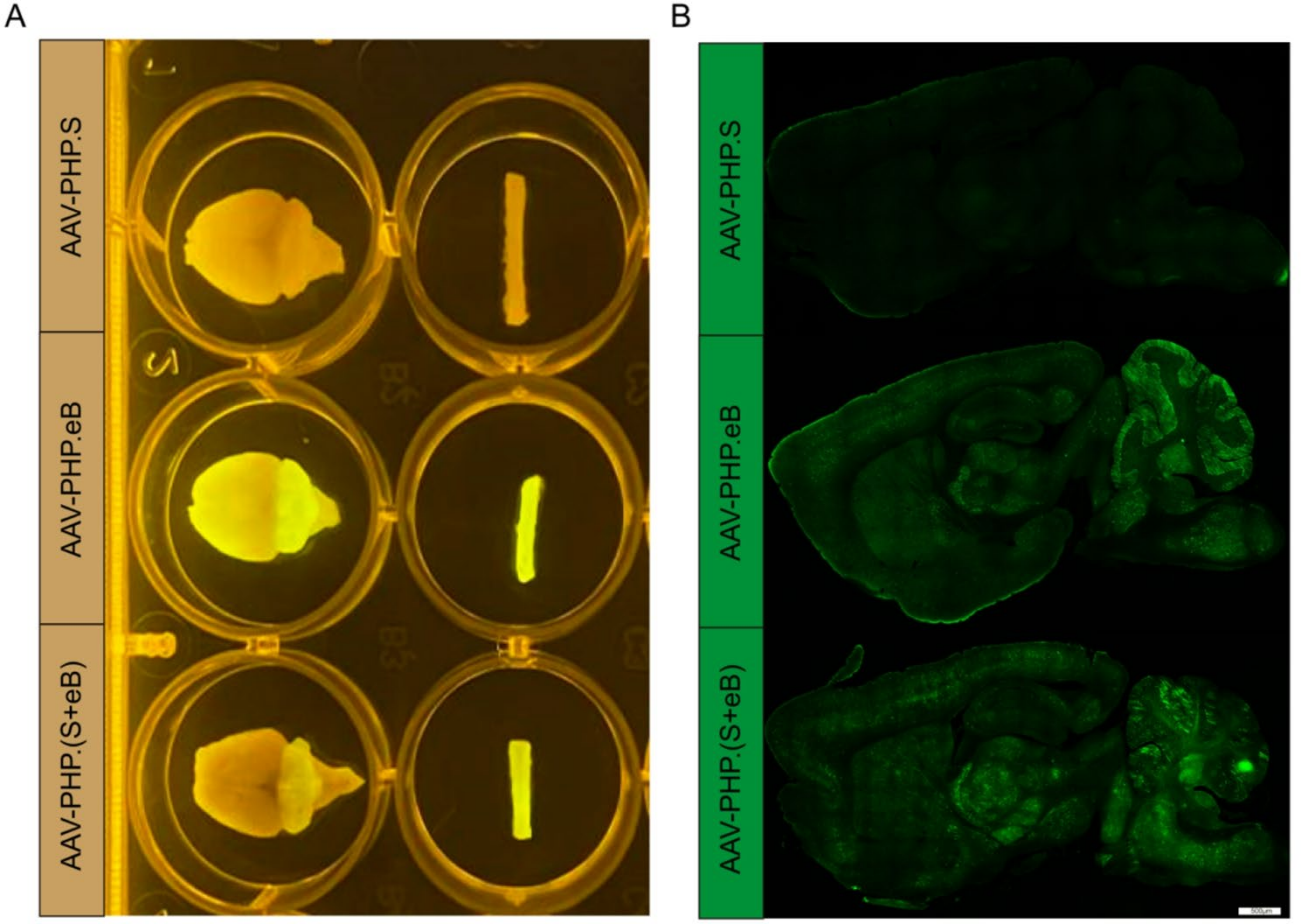
Systemic delivery of AAV-PHP.(S+eB) mediates widespread transduction in the CNS. **A** AAV was packaged into the indicated capsid and intravenously injected into 8-week-old mice at 4 × 10^11^ VG per mouse (AAV-PHP.S, AAV-PHP.eB and AAV-PHP.(S+eB)). Representative whole-brain fluorescence images after 4 weeks of expression. **B** Representative confocal images of EGFP fluorescence from sagittal brain sections for the indicated capsids. Scale bars for B are 500 μm

Systemic administration of AAV-PHP.(S + eB) (intravenous injection of 4 × 10^11^ VG per mouse via tail vein) into 8-week-old mice resulted in widespread transgene expression in the CNS. Four weeks post-injection, gross fluorescence imaging of brain and spinal cord, as well as immunofluorescence analysis of brain sections, revealed broad and robust EGFP expression across multiple CNS regions. AAV-PHP.(S + eB) mediated extensive cellular transduction in both brain and spinal cord, which is comparable to that of AAV-PHP.eB and much higher than AAV-PHP.S alone(Fig. 1A and B).

### AAV-PHP.(S + eB) transduce neurons more effectively than non-neuronal cells across the CNS

To further evaluate the transgene (EGFP) expression profiles of AAV-PHP.(S + eB) compared with AAV-PHP.eB and AAV-PHP.S in the CNS, we performed immunofluorescence staining on spinal cord and brain sections. Neurons (NeuN), astrocytes (GFAP), microglia (IBA1), and oligodendrocytes (SOX10) were labeled, and the proportion of EGFP-positive cells co-localizing with each marker was quantified to determine cell-type transduction efficiency.

**Fig. 2:**
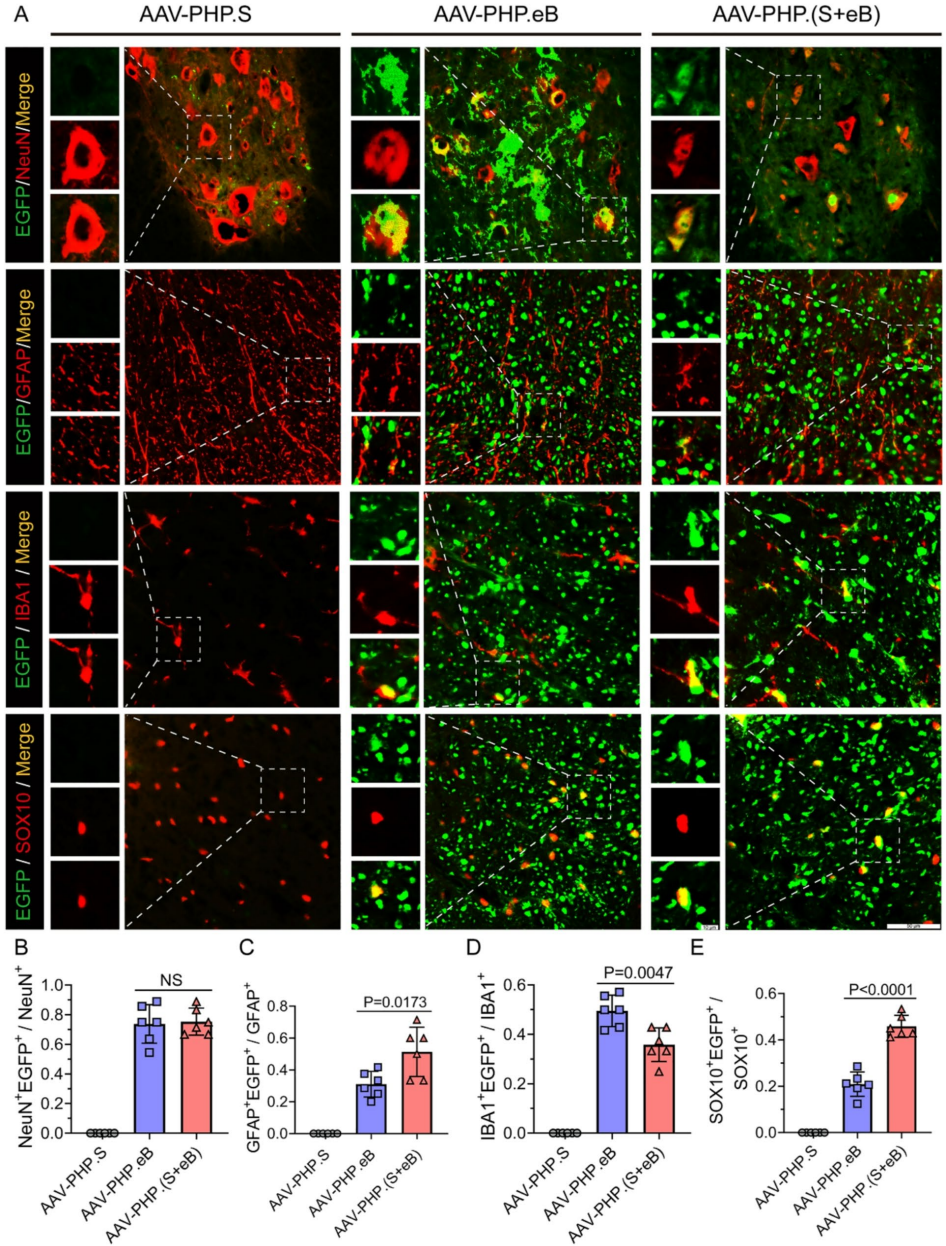
Spinal cord transduction by AAV-PHP.(S+eB). **A** Representative immunofluorescence images of spinal cord sections showing EGFP (green) and cell-type markers (red): NeuN (neurons), GFAP (astrocytes), SOX10 (oligodendrocytes), IBA1 (microglia). Scale bar, 50 μm. **B**–**E** Quantification of transduction efficiency showing that AAV-PHP.(S+eB) retains neuronal tropism comparable to AAV-PHP.eB, with enhanced transduction of oligodendrocytes and astrocytes but reduced microglial transduction. AAV-PHP.S exhibited minimal transduction across all cell types. Data are mean ± SEM (n = 6)

**Fig. 3:**
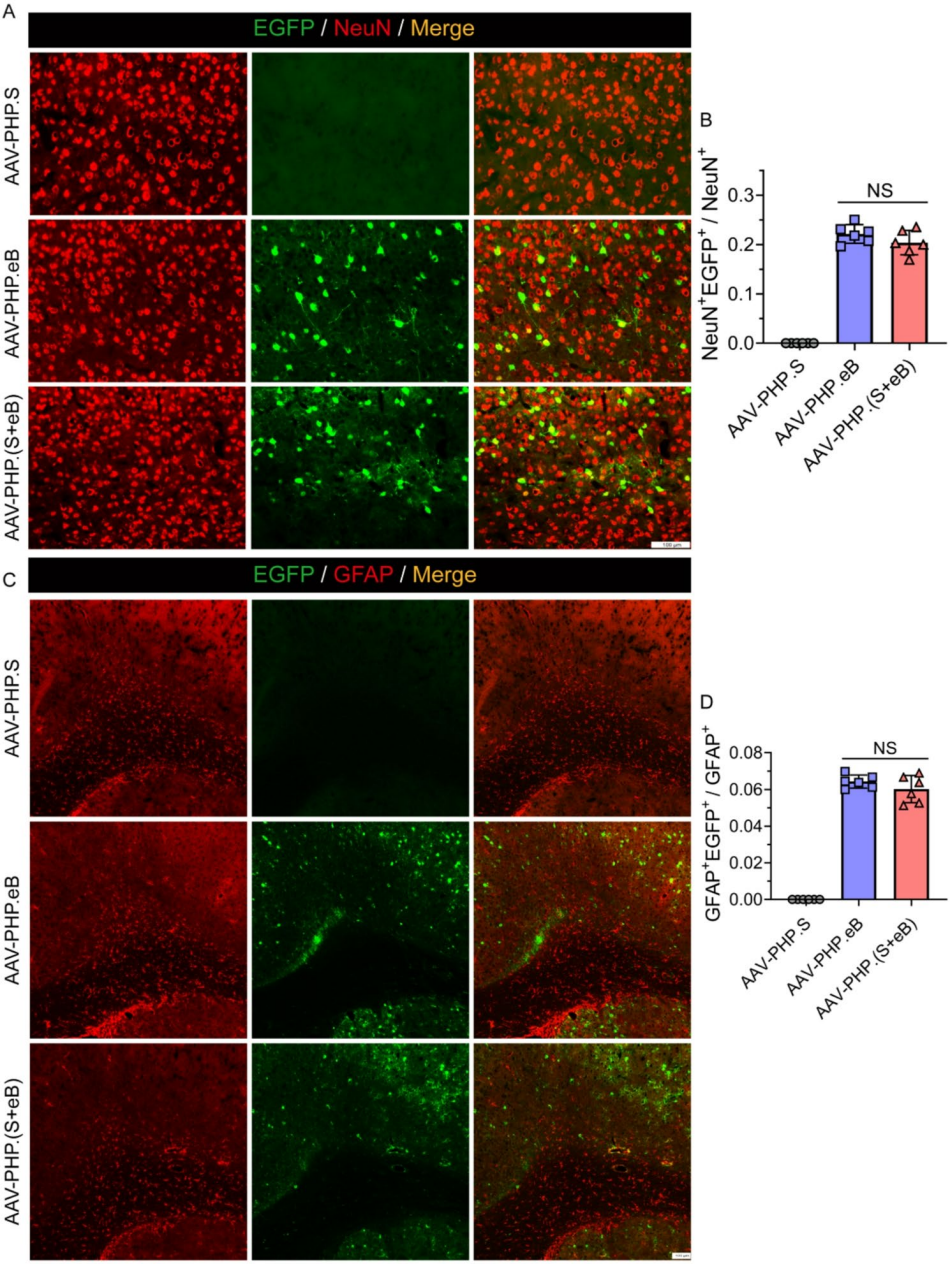
Brain transduction in neurons and astrocytes. **A**, **B** Representative images and quantification of EGFP expression (green) in NeuN⁺ neurons (red). **C**, **D** Representative images and quantification in GFAP⁺ astrocytes (red). AAV-PHP.(S+eB) and AAV-PHP.eB showed comparable neuronal transduction, with both vectors mediating only limited astrocytic transduction. Scale bar, 100= μm. Data are mean ± SEM (n = 6)

**Fig. 4:**
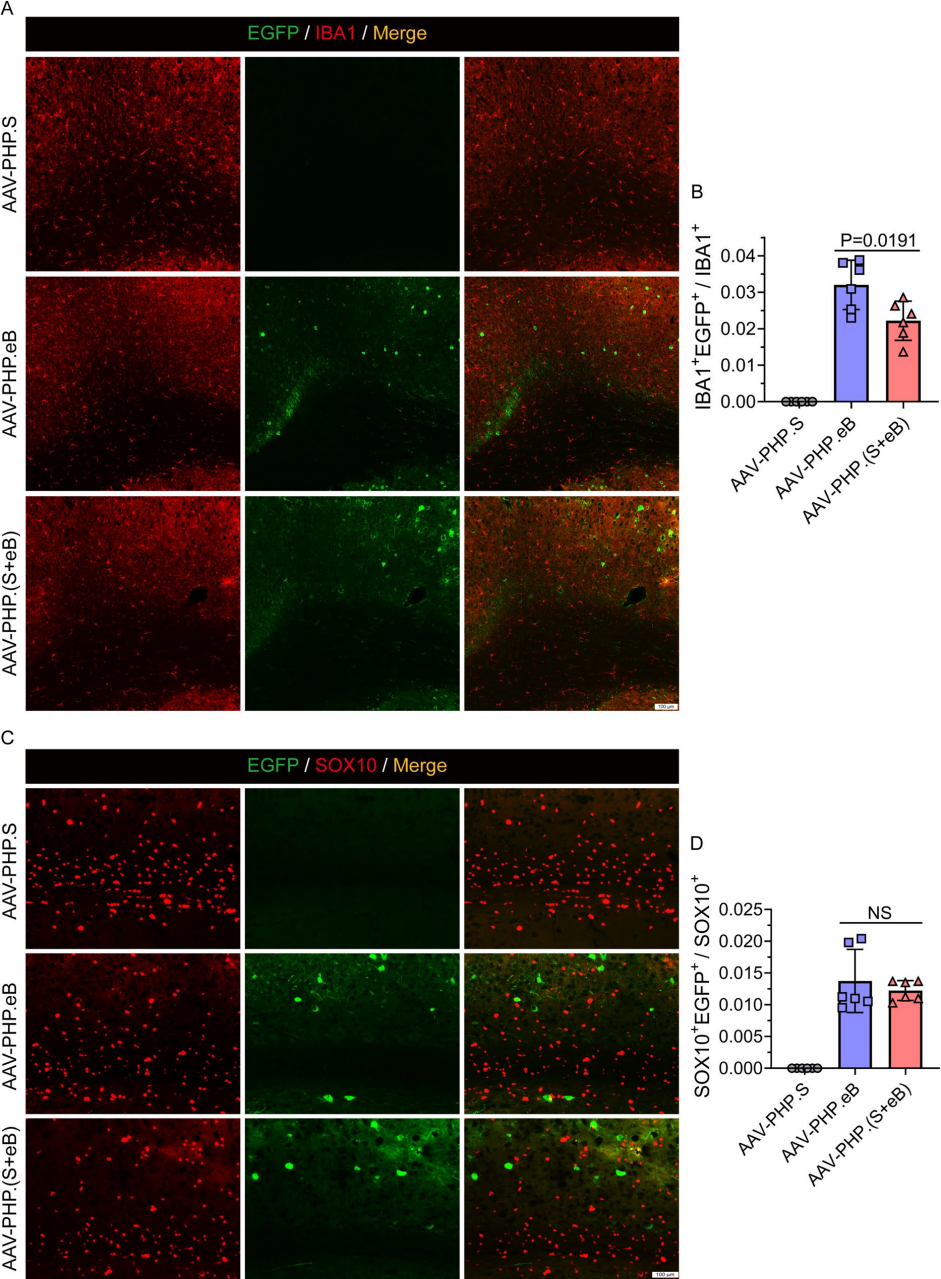
Brain transduction in microglia and oligodendrocytes. **A**, **B** EGFP expression in IBA1⁺ microglia (red). **C**, **D** EGFP expression in SOX10⁺ oligodendrocytes (red). Both AAV-PHP.(S+eB) and AAV-PHP.eB exhibited minimal transduction in microgliaand oligodendrocytes, while AAV-PHP.S showed negligible activity. Scale bar, 100 μm. Data are mean ± SEM (n = 6)

While AAV-PHP.S displayed consistently low transduction efficiencies across all cell types in both spinal cord and brain tissues, AAV-PHP.eB and AAV-PHP.(S + eB) showed similar levels of transduction. In the spinal cord, analysis of transduction efficiency revealed that in NeuN⁺ neurons, AAV-PHP.eB and AAV-PHP.(S + eB) achieved mean efficiencies of 73% and 75%, respectively (Fig. 2A and B). In GFAP⁺ astrocytes, the mean efficiencies were 31% and 51% (Fig. 2C), while in Iba1⁺ microglia the efficiencies were 50% and 36%, respectively (Fig. 2D). In SOX10⁺ oligodendrocytes, the mean efficiencies were 20% and 45% (Fig. 2E). In the brain, the corresponding efficiencies were as follows: NeuN⁺ neurons, 22% for AAV-PHP.eB and 20% for AAV-PHP.(S + eB) (Fig. 3A and B); GFAP⁺ astrocytes, 6.4% and 6.0% (Fig. 3C and D); IBA1⁺ microglia, 3.2% and 2.2% (Fig. 4A and B); and SOX10⁺ oligodendrocytes, 1.4% and 1.2% (Fig. 4C and D).

These results demonstrate that in NeuN⁺ neurons, AAV-PHP.(S + eB) and AAV-PHP.eB exhibited comparable transduction efficiencies in both spinal cord and brain. In glial cells, AAV-PHP.(S + eB) mediated significantly higher transduction of GFAP⁺ astrocytes and SOX10⁺ oligodendrocytes, but lower transduction of IBA1⁺ microglia in the spinal cord compared with AAV-PHP.eB. In the brain, both vectors showed limited glial transduction. Overall, these findings indicate that AAV-PHP.(S + eB) retains CNS transduction properties similar to those of AAV-PHP.eB, suggesting that the mixed-capsid packaging strategy preserves CNS tropism.

### AAV-PHP.(S + eB) has efficient transduction of DRG neurons in PNS

**Fig. 5:**
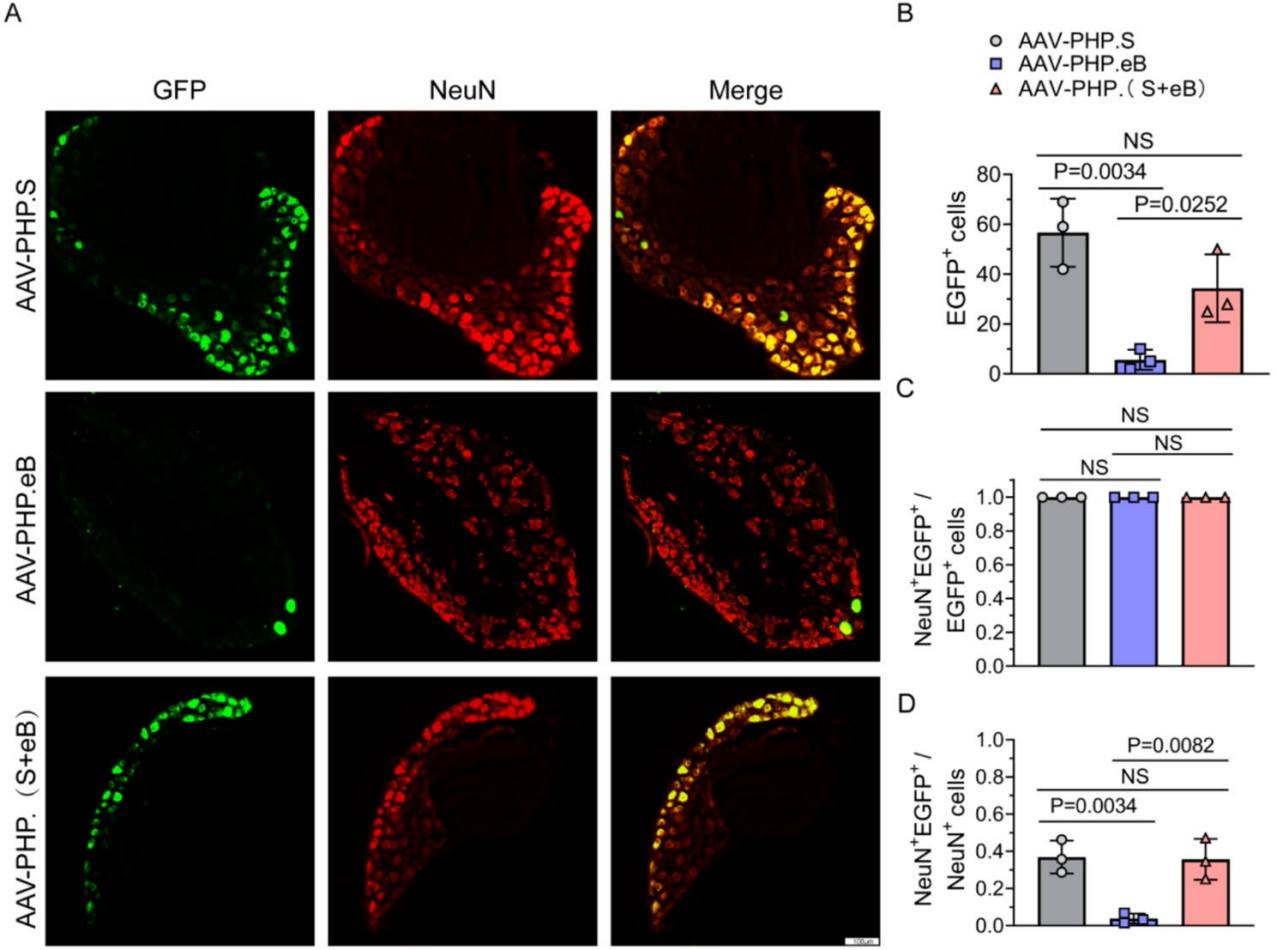
DRG transduction by AAV-PHP.(S+eB). **A** Representative immunofluorescence of DRG sections showing EGFP expression (green) and NeuN labeling (red); yellow indicates co-localization. Scale bar, 50 μm. **B**–**D** Quantification of EGFP⁺ cells, NeuN⁺EGFP⁺/EGFP⁺ cells, and NeuN⁺EGFP⁺/NeuN⁺ cells, demonstrating that AAV-PHP.(S+eB) achieves neuronal transduction comparable to AAV-PHP.S and significantly higher than AAV-PHP.eB. Data are mean ± SEM (n = 3)

We next assessed the transduction efficiency of AAV-PHP.(S + eB) in the peripheral nervous system (PNS). The dorsal root ganglion (DRG), a critical relay for peripheral sensory signaling and a key structure linking the PNS to the central nervous system (CNS), was used as a classical model for evaluating AAV-mediated peripheral delivery. Neurons were immunolabeled with the neuronal marker NeuN, and the proportion of EGFP/NeuN double-positive cells was quantified as a measure of transduction efficiency. AAV-PHP.S achieved an average transduction efficiency of ~ 35%, AAV-PHP.(S + eB) reached ~ 36%, whereas AAV-PHP.eB transduced only ~ 3% of DRG neurons (Fig. 5A–D). These results demonstrate that AAV-PHP.(S + eB) achieves DRG transduction efficiency comparable to AAV-PHP.S and markedly surpasses that of AAV-PHP.eB. Together with the CNS data, these findings indicate that the mosaic packaging strategy allows AAV-PHP.(S + eB) to preserve the CNS tropism of AAV-PHP.eB while simultaneously acquiring the robust DRG tropism of AAV-PHP.S, suggesting that this engineered capsid combines the tissue-specific targeting properties of both parental variants.

### AAV-PHP.(S + eB) Haslower liver tropism than AAV-PHP.eB and AAV-PHP.S

In the context of gene therapy, off-target transduction in non-target organs is a critical concern. Previous studies have shown that AAV-PHP.eB exhibits relatively low hepatotoxicity, whereas AAV-PHP.S, although less hepatotoxic than AAV9, induces higher liver transduction than AAV-PHP.eB. To assess the hepatic off-target transduction of the newly engineered AAV-PHP.(S + eB), we systematically examined EGFP expression in mouse liver. Comparative analysis of EGFP fluorescence intensity revealed that, at equivalent injection doses, AAV-PHP.(S + eB) mediated significantly lower hepatic transduction compared with both AAV-PHP.eB and AAV-PHP.S. Quantification of mean fluorescence intensity showed that signal strength for AAV-PHP.(S + eB) was approximately 45% and 59% relative to the parental vectors. These findings indicate that AAV-PHP.(S + eB) achieves lower hepatocyte transduction efficiency, suggesting a potentially improved liver safety profile.

## Discussion

In this study, we generated a mosaic AAV vector, AAV-PHP.(S + eB), by mixing the Rep/Cap plasmids of AAV-PHP.eB and AAV-PHP.S during virus packaging. Analysis of EGFP expression across tissues demonstrated that AAV-PHP.(S + eB) retains the CNS tropism characteristic of AAV-PHP.eB while achieving dorsal root ganglia (DRG) transduction efficiency comparable to AAV-PHP.S. Moreover, this vector exhibits reduced liver transduction, indicating that the mixed packaging strategy holds potential for achieving multi-tissue targeted gene delivery.

A major limitation of existing AAV variants is their restricted tissue specificity, often necessitating separate vectors to target the CNS and PNS. Previous studies have shown that systemic administration of AAV-PHP.eB results in efficient CNS transduction in C57BL/6J mice, primarily attributed to its enhanced ability to cross the BBB [10, 19, 20]. In contrast, AAV-PHP.S efficiently targets DRG and other peripheral sensory neurons but demonstrates poor CNS transduction [10, 21]. Our results reveal that AAV-PHP.(S + eB) preserves the broad CNS transduction profile of AAV-PHP.eB while achieving DRG transduction levels comparable to AAV-PHP.S. This dual targeting capability may be particularly beneficial for neurological disorders involving both central and peripheral neural circuits, such as amyotrophic lateral sclerosis and peripheral neuropathies. Importantly, it enables simultaneous CNS and PNS targeting without increasing viral injection doses, thereby minimizing off-target effects and immune responses associated with higher vector loads [22] .

Immunofluorescence analyses showed that in both spinal cord and brain tissues, transduction efficiency of AAV-PHP.(S + eB) in neurons (NeuN-positive cells) was similar to that of AAV-PHP.eB [10, 23], while transduction in non-neuronal cells remained low, consistent with prior reports of predominantly neuronal tropism of AAV-PHP.eB in adult mice. In DRG, AAV-PHP.eB displayed minimal transduction efficiency [10], whereas AAV-PHP.S reached approximately 35%. Notably, AAV-PHP.(S + eB) achieved around 36% transduction efficiency, nearly matching AAV-PHP.S. This improvement likely arises from capsid heterogeneity generated during vector production, resulting in a viral population that retains both BBB-crossing and PNS-targeting capabilities.

The AAV capsid consists of 60 overlapping subunits of VP1, VP2, and VP3, with VP3 primarily determining serotype-specific receptor binding characteristics [24, 25]. Alterations in the amino acid sequence of surface-exposed regions of VP3 can affect tissue tropism, receptor interactions, and immunogenicity [26–28]. When two (or more) distinct Rep/Cap plasmids are co-transfected during packaging, the 60 subunits are randomly assembled from VP proteins derived from different serotypes, generating mosaic capsids. Previous studies have proposed the use of mixed or mosaic capsids to expand tropism spectra [29]. Our data provide in vivo evidence supporting the feasibility of this approach.

In summary, our findings demonstrate that mosaic capsid packaging is a powerful strategy to overcome the inherent tissue-specific limitations of conventional AAV variants. By combining distinct capsid serotypes, AAV-PHP.(S + eB) effectively achieves dual targeting of both CNS and PNS, while simultaneously reducing off-targeting of liver. This balance of specificity, efficiency, and safety expands the versatility of the AAV capsid toolkit and provides a flexible platform for developing tailored gene therapies. Such an approach is particularly relevant for neurological disorders involving both central and peripheral nervous systems, where simultaneous modulation of multiple tissues is required. Our study implicated that mosiac AAV composed of various types of CNS specific capsids has a potential for highly efficient CNS targeting, which is still a challenge for efficient transduction of primates.

## Methods

### Animals

Both sexes of B6 mice ages 2 months were used in this study. All experimental procedures were carried out in accordance with the approval of the Institutional Animal Ethics Committee of Hangzhou Normal University and complied with the requirements of the Regulations for the Administration of Laboratory Animals.

### Viruses production and injections

AAV2/PHP.S-CMV-bGI-EGFP-WPRE-pA (S0263-PS-H50), AAV2/PHP.eB-CMV-bGI-EGFP-WPRE-pA (S0263-PeB-H50) were purchased from Shanghai Taitool Bioscience Co., Ltd. pUCmini-iCAP-PHP.eB (Addgene plasmid #103005; http://n2t.net/addgene:103005; RRID: Addgene_103005) and pUCmini-iCAP-PHP.S (Addgene plasmid #103006; http://n2t.net/addgene:103006; RRID: Addgene_103006) were gifts from Viviana Gradinaru [10]. HEK 293T cells were transfected with pHelper, reporter cargo, and either pUCmini-iCAP-PHP.eB or pUCmini-iCAP-PHP.S at 1:1:1 ratio for AAV production. And equal amount of pUCmini-iCAP-PHP.eB and pUCmini-iCAP-PHP.S were co-transfected for mosaic AAV production. Three days after transfection, culture medium was collected, and centrifuged at 3000 g for 10 min to remove debris. And 40% PEG8000 was added to the supernatant to achieve a final concentration of 8%. The mixture was then gently agitated and incubated at 4 °C for 2 h to facilitate the precipitation of virus particles. The resulting viral pellet was collected by centrifugation (3000 g for 30 min), and the supernatant was discarded. The pellet was resuspended in PBS and combined with the supernatant of cell lysates. Following centrifugation at 3,000 g for 30 min, the supernatant was collected and subsequently filtered to obtain the virus concentrate. The final virus was obtained through two rounds of CsCl density gradient centrifugation followed by ultrafiltration. Viral titers (virus genome/ml, vg/ml) were quantified by qPCR using pWPRE primers: forward (CCTTTCCGGGACTTTCGCTTT) and reverse (GCAGAATCCAGGTGGCAACA). Adult mice at 2 months of age were administered at a dose of 4 × 10^11^ vg/mouse through tail-vein intravenous injection.

### Tissue preparation and immunohistochemistry

Seven days post-injection, mice were anesthetized and then transcardially perfused with PBS followed by 4% cold paraformaldehyde (PFA). Brain, spinal cord, DRG, and liver tissue were dissected and then fixed overnight at 4 °C in 4% PFA. Two days after the tissues were transferred to a 30% sucrose solution, they were embedded in an optimal cutting temperature compound (OCT) and sectioned to a thickness of 14 μm.

After antigen retrieval in a citrate buffer at 85–98 °C for 25 min, the sections were rinsed three times with 1×PBS. After being incubated in a blocking solution containing 10% goat serum and 0.1% Triton X − 100 at room temperature for 1 h, the sections were further incubated with primary antibodies in the same blocking solution at 4 °C overnight. The antibody used in this study were rabbit anti-SOX10 (OB-PRB053, Oasis Biofarm), rabbit anti-NeuN (OB-PRB039, Oasis Biofarm), rabbit anti-IBA1 (OB-PRB029, Oasis Biofarm), rabbit anti-GFAP (OB-PRB005, Oasis Biofarm) and guinea pig anti-EGFP (OB-PGP003, Oasis Biofarm).

On the following day, tissue sections were washed three times with 1× PBS. The sections were then incubated with secondary antibodies for 2 h at room temperature. The secondary antibody used were Goat anti-Rabbit IgG (H + L), Alexa Fluor 594 (A11012; Thermo Fisher), Goat anti-Guinea Pig IgG (H + L), Alexa Fluor 488 (A11073; Thermo Fisher). After a single wash, the nuclei were counterstained with DAPI. Finally, the tissue sections were mounted on glass slides using Mowiol mounting medium (MMM). Fluorescence imaging of the samples was carried out using a ZEISS or Leica epifluorescence microscope.

### Statistical analyses

For the quantification of dual-positive cell populations, manual counting was performed to determine the number of double-positive cells in identically selected and standardized regions of interest (ROIs) across all experimental groups to ensure accuracy. Fluorescence intensity was analyzed using ImageJ software. Data processing was carried out using GraphPad Prism software for statistical analysis. For comparisons among experimental groups, one - way or two - way analysis of variance (ANOVA) was used. All data are presented as mean ± standard error of the mean (SEM). Statistical significance was defined as *p* < 0.05.

**Fig. 6:**
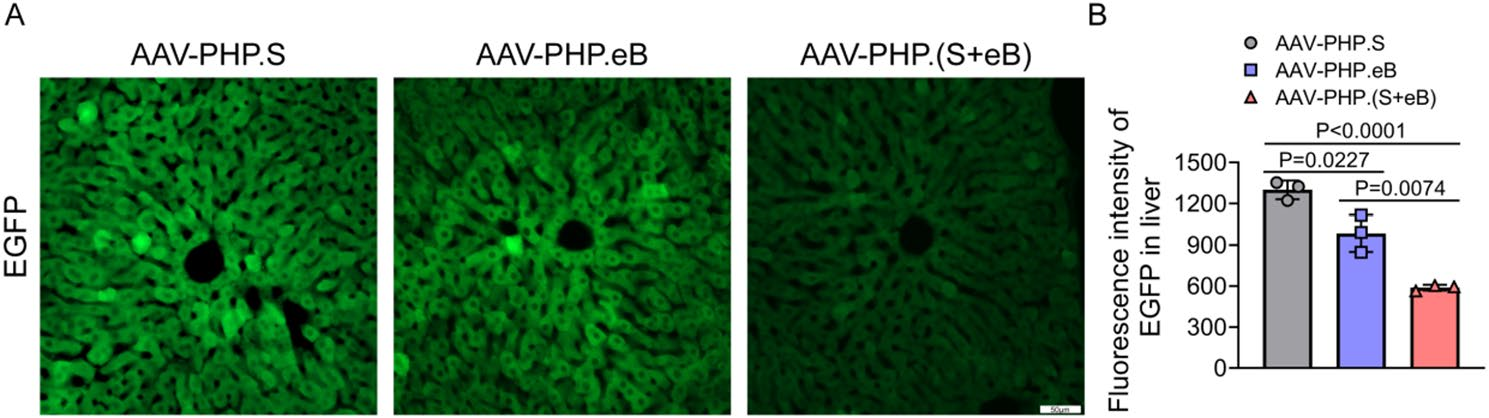
Reduced Liver Transduction of AAV-PHP.(S+eB). **A** Representative EGFP fluorescence images of liver sections from mice injected with AAV-PHP.eB, AAV-PHP.S, or AAV-PHP.(S+eB) at equivalent doses. Scale bar, 50 μm. **B** Quantification of mean EGFP fluorescence intensity, showing that AAV-PHP.(S+eB) mediates significantly lower hepatic transduction compared with both parental capsids. Data are mean ± SEM (n = 3)

## Data Availability

No datasets were generated or analysed during the current study.
